# ASPM promotes glioblastoma growth by regulating G1 restriction point progression and Wnt-β-catenin signaling

**DOI:** 10.18632/aging.102612

**Published:** 2020-01-06

**Authors:** Xin Chen, Lijie Huang, Yang Yang, Suhua Chen, Jianjun Sun, Changcheng Ma, Jingcheng Xie, Yongmei Song, Jun Yang

**Affiliations:** 1 Department of Neurosurgery, Peking University Third Hospital, Beijing 100191, China; 2 Beijing Neurosurgical Institute, Capital Medical University, Beijing 100070, China; 3 Department of General Surgery, The Fourth Hospital of Hebei Medical University, Shijiazhuang 050011, China; 4 State Key Laboratory of Molecular Oncology, National Cancer Center/Cancer Hospital, Chinese Academy of Medical Sciences and Peking Union Medical College, Beijing 100021, China

**Keywords:** ASPM, bioinformatical analysis, cell cycle, glioblastoma, Wnt-β-catenin signaling

## Abstract

Increasing evidence has indicated that the disorganized expression of certain genes promotes tumour progression. In this study, we elucidate the potential key differentially expressed genes (DEGs) between glioblastoma (GBM) and normal brain tissue by analysing three different mRNA expression profiles downloaded from the Gene Expression Omnibus (GEO) database. DEGs were sorted, and key candidate genes and signalling pathway enrichments were analysed. In our analysis, the highest fold change DEG was found to be abnormal spindle-like microcephaly associated (ASPM). The ASPM expression pattern from the database showed that it is highly expressed in GBM tissue, and patients with high expression of ASPM have a poor prognosis. Moreover, ASPM showed aberrantly high expression in GBM cell lines. Loss-of-function assay indicated that ASPM enhances tumorigenesis in GBM cells *in vitro*. Xenograft growth verified the oncogenic activity of ASPM *in vivo*. Furthermore, downregulation of ASPM could arrest the cell cycle of GBM cells at the G0/G1 phase and attenuate the Wnt/β-catenin signalling activity in GBM. These data suggest that ASPM may serve as a new target for the therapeutic treatment of GBM.

## INTRODUCTION

GBM is the most frequent and aggressive type of primary malignant brain tumour, with an incidence of 3–4/100,000 [1]. It represents 16% of all brain tumours and 54% of all gliomas [2]. Although these tumours may develop at all ages, the incidence increases with age, with the peak incidence being in the fifth or sixth decade [3]. To date, therapeutic options consist of microsurgery and treatment with radiotherapy combined with adjuvant chemotherapy with an alkylating agent, temozolomide (TMZ). Although microsurgical resection can greatly reduce tumour volume, complete excision is almost impossible because of the infiltrative nature of the tumours. Although there have been a number of advances in the radiotherapy/TMZ treatment of GBM, the prognosis for this type of cancer is still very poor. For patients with newly diagnosed GBM, the progression-free survival (PFS) is approximately 7–8 months, the median survival is approximately 15-18 months, and approximately 10% of patients go on to be five-year survivors [4–6]. Despite many efforts, GBM invariably recurs and leads to a fatal outcome.

Microarray is a molecular biological technology that accompanied the development of the Human Genome Project (HGP). A large number of known sequence probes are integrated on the same substrate, and then the labelled target nucleotides are hybridized with probes at specific locations on the microarray. By detecting hybridization signals, a large amount of gene information in biological cells and tissues was analysed [7]. With the large-scale application of microarray technology over the years, there have been many GBM microarray data stored in public databases. However, the results are always limited or generated from a single cohort study, which cannot identify reliable target genes. Thus, integrating bioinformatics analysis with expression profiling technology may overcome these shortcomings.

To better understand the influence of key DEGs on the molecular pathogenesis of GBM, in this study, we downloaded three GBM-related original microarray datasets, GSE14818, GSE22866 and GSE50161, from the GEO database (https://www.ncbi.nlm.nih.gov/geo), consisting of a total of 104 samples, with 88 cases of GBM samples and 16 cases of normal samples [8, 9]. DEGs in GBM and normal brain samples were screened using R software. We developed Gene Ontology and pathway enrichment analysis for screening DEGs with DAVID (https://david.ncifcrf.gov/) and KEGG PATHWAY (http://www.genome.jp/kegg) in GBM. Subsequently, the DEGs identified in the present study were validated using microarray data from The Cancer Genome Atlas (TCGA) database. Finally, one of the most upregulated genes, ASPM, which has the highest fold change among DEGs, was further studied and identified *in vitro* and *in vivo.* The present study explored key DEGs and key pathways in GBM and identified ASPM as a promising therapeutic target of GBM.

## RESULTS

### 362 candidate key genes were identified from GEO microarray datasets

**Figure 1:**
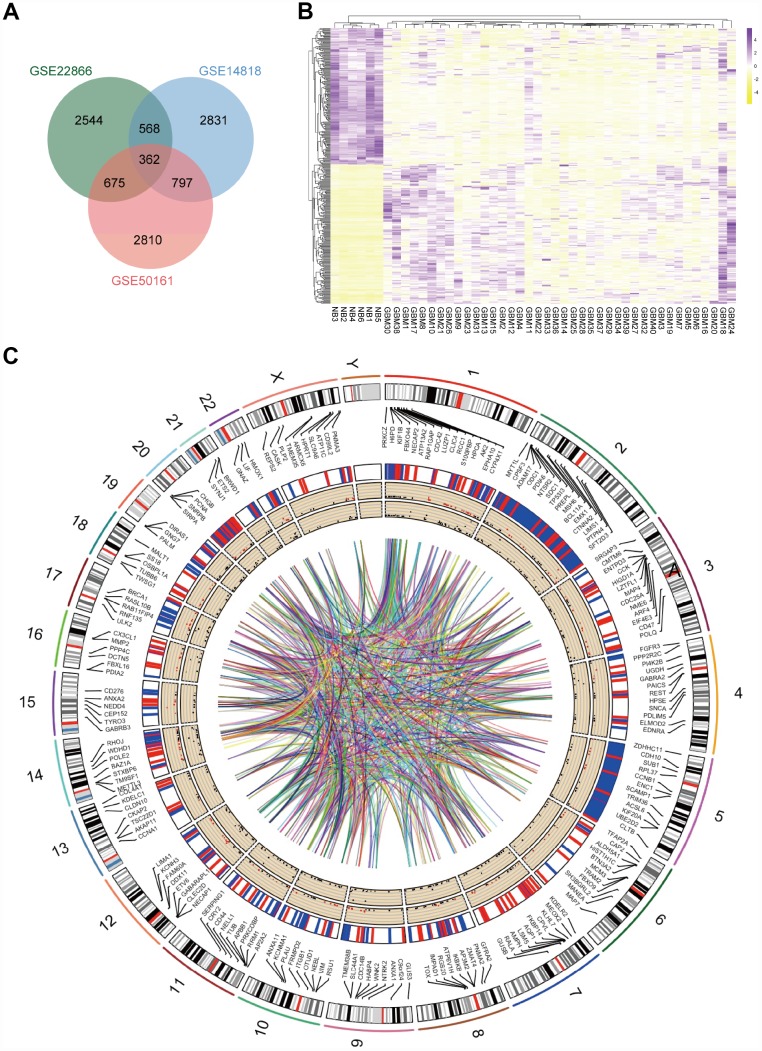
**Identification of DEGs in mRNA datasets GSE14818, GSE22866 and GSE50161.** (**A**) Venn diagram presents overlapping relationships, and 362 DEGs were identified. (**B**) Cluster analysis of DEGs using data profile GSE22866 as a reference. Data are presented as a heat map. (**C**) Circos plot showing DEGs on human chromosomes. From the outside in, the first layer of the circos plot is a chromosome map of the human genome, black and white bars are chromosome cytobands, and red bars represent centromeres. Due to limited space, some of the DEGs are labelled in the second circle. In the third layer, up- and downregulated DEGs are marked in red and blue, respectively. The fourth layer represents the fold change of DEGs with fold change ≥ 2.0. The innermost circle indicates the DEGs with p-value < 0.05. The network in the centre of the plot represents the core network.

GBM and normal brain tissue gene expression profiles from GSE14818, GSE22866 and GSE50161 were obtained from NCBI-GEO. The GSE14818 microarray data had 10 GBM tissues and 2 normal brain tissues from Germany, and 5300 DEGs were obtained. Among them, 2006 downregulated genes and 3249 upregulated genes were identified. The GSE22866 dataset had 40 GBM tissues and 6 normal brain tissues from France, and overall, 7012 DEGs were screened from the dataset, including 5061 upregulated genes and 1951 downregulated genes. Additionally, 6627 DEGs, including 2849 upregulated genes and 3733 downregulated genes, were screened from the GSE50161 dataset, which had 34 GBM tissues and 8 normal brain tissues from America. A total of 362 consistently expressed genes were identified from the three datasets (Figure 1A) after integrated bioinformatical analysis, including 185 upregulated genes and 177 downregulated genes in the GBM tissues compared to normal brain tissues. To show the significant differential distribution of the 362 DEGs, a heat map of the up- and downregulated DEGs was drawn using the data profile from GSE22866 as a reference (Figure 1B). DEGs on human chromosomes are shown in the circos plot (Figure 1C).

### Validation of the DEGs in the TCGA GBM dataset

**Figure 2:**
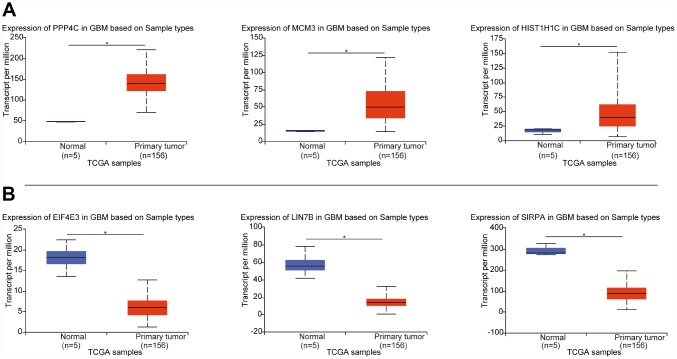
**The expression of DEGs in the three datasets from this study in the TCGA dataset.** (**A**) Expression of the top 3 upregulated DEGs from TCGA GBM. (**B**) Expression of the top 3 downregulated DEGs from TCGA GBM. *p < 0.05 indicates a significant difference.

To certify the reliability of the 362 DEGs identified from the 3 datasets, we downloaded the TCGA GBM dataset, which provides extensive genetic studies of human gene expression and specific disease associations. We found that the 148 upregulated DEGs found in this study were also significantly expressed in the GBM dataset, and the 124 downregulated genes found in this study were also significantly expressed in GBM (Figure 2 and Supplementary Table 1). The overall consistency of the DEGs was 75.13%, indicating that the results of our candidate genes were reliable.

### GO term enrichment analysis of DEGs showed most of the DEGs were significantly enriched in transport, binding, cell parts and cell cycle

**Figure 3:**
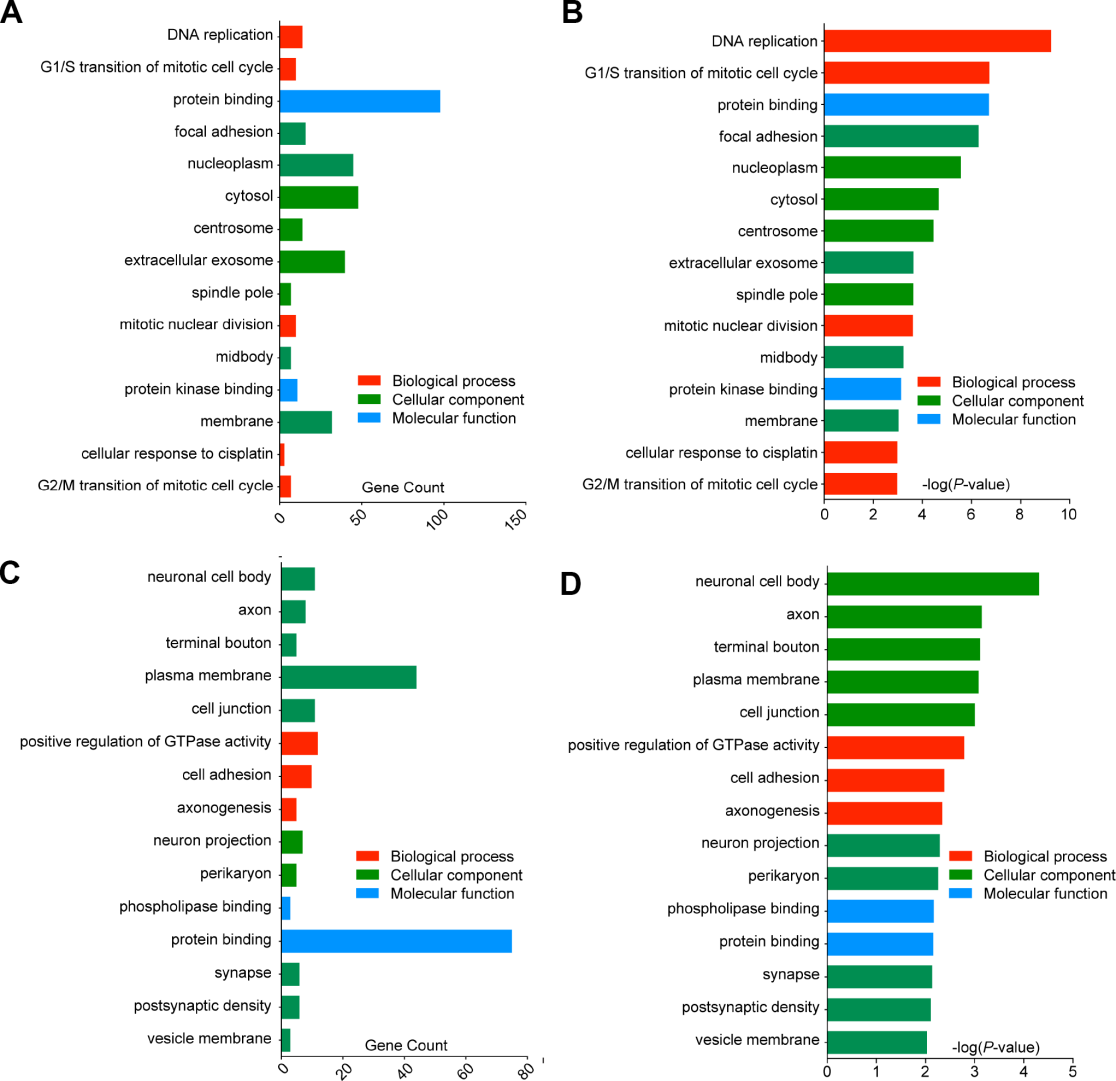
**Gene Ontology analysis and significantly enriched GO terms of DEGs in GBM. The top 15 enriched DEGs of each group are shown (p < 0.05).** (**A**) GO analysis classified the upregulated DEGs into 3 groups (molecular function, biological process and cellular component), gene counts of each GO terms were showed. (**B**) Significantly enriched GO terms of upregulated DEGs in GBM based on their p-value. (**C**) GO analysis classified the downregulated DEGs into 3 groups, gene counts of each GO terms were showed. (**D**) Significantly enriched GO terms of downregulated DEGs in GBM based on their p-value.

Functions and pathway enrichment of the candidate DEGs in GBM identified from an integrated analysis of microarray data were analysed using multiple online databases, including DAVID and KEGG PATHWAY. GO functional enrichments of up- and downregulated genes were performed with DAVID, and enriched GO terms with *P*-values < 0.05 were obtained. GO analysis of DEGs was divided into three functional groups, including biological processes (BP), cell composition (CC) and molecular function (MF). The enriched GO terms for the DEGs are presented in Figure 3. For the upregulated DEGs, the top 3 enriched GO terms for each group were DNA replication, G1/S transition of mitotic cell cycle and mitotic nuclear division in the BP category; focal adhesion, nucleoplasm and cytosol in the CC category; and protein binding, protein kinase binding and cysteine-type endopeptidase activity in the MF category (Figure 3A, 3B). Similarly, for the downregulated DEGs, the top 3 enriched GO terms for each group were positive regulation of GTPase activity, cell adhesion and axonogenesis in the BP category; neuronal cell body, axon and terminal bouton in the CC category; and phospholipase binding, protein binding and actin binding in the MF category (Figure 3C, 3D). These results showed that most of the DEGs were significantly enriched in transport, binding, cell parts and cell cycle.

### Signalling pathway enrichment analysis showed that DEGs had the most common pathways in cell cycle

**Figure 4:**
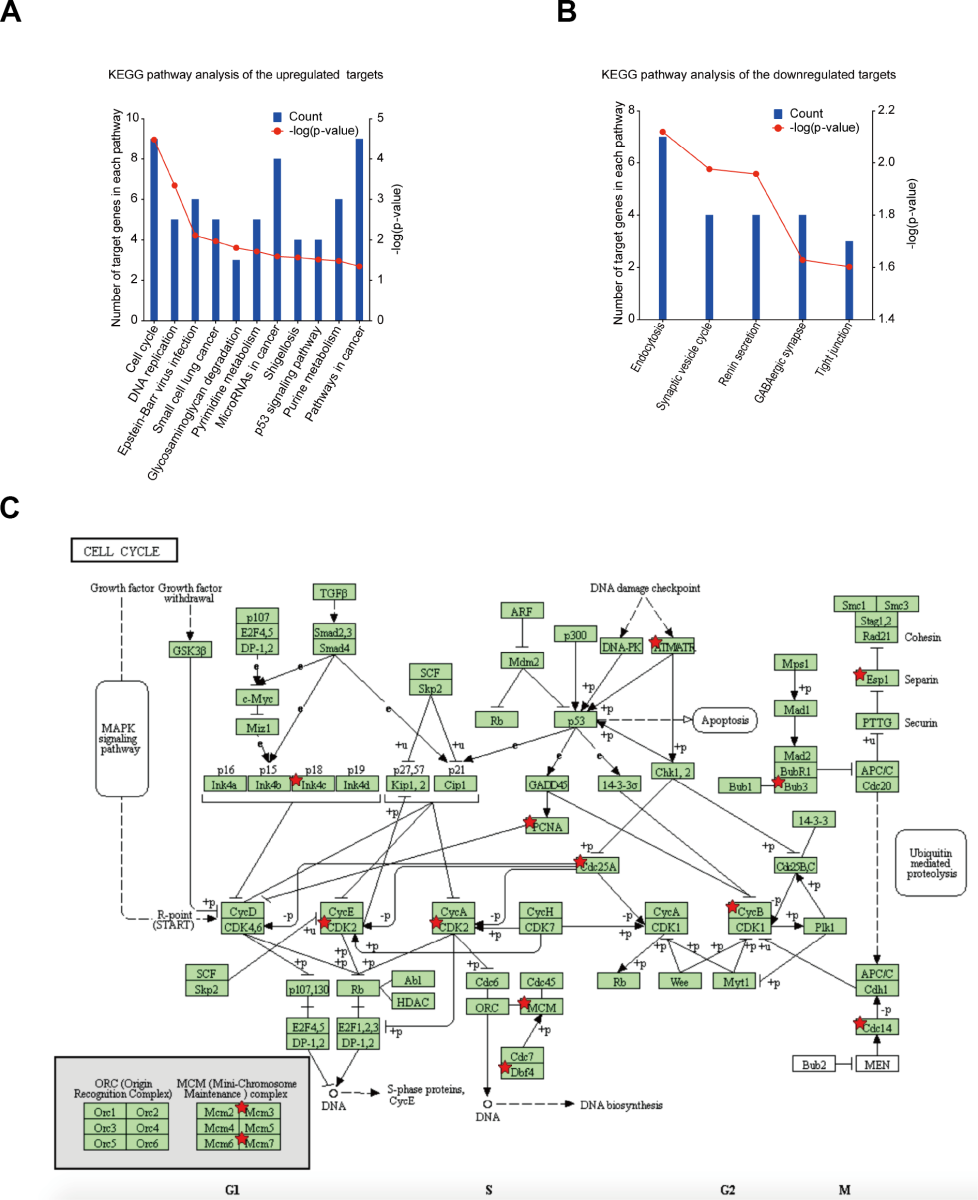
**Significantly enriched pathway terms of DEGs in GBM.** (**A**) KEGG pathway enrichment analysis of upregulated mRNAs (p < 0.05). (**B**) KEGG pathway enrichment analysis of downregulated mRNAs (p < 0.05). (**C**) KEGG pathway annotations of the cell cycle pathway. Red star-marked nodes are associated with significant genes.

DEG functional and signalling pathway enrichment was performed using the DAVID and KEGG PATHWAY online websites. The enriched KEGG pathways for the upregulated DEGs include cell cycle, DNA replication, Epstein-Barr virus infection, small cell lung cancer, glycosaminoglycan degradation, pyrimidine metabolism, microRNAs in cancer, shigellosis, p53 signalling pathway, purine metabolism and pathways in cancer (Figure 4A). For the downregulated DEGs, the enriched KEGG pathways include endocytosis, synaptic vesicle cycle, renin secretion, GABAergic synapse and tight junction (Figure 4B). The cell cycle pathway was considered to be the most central functions because it shared the most connected genes and the lowest *p* value. Signalling pathway analysis showed that DEGs had the most common pathways in cell cycle (Figure 4C).

### ASPM is highly expressed in human glioma tissues and cells

**Figure 5:**
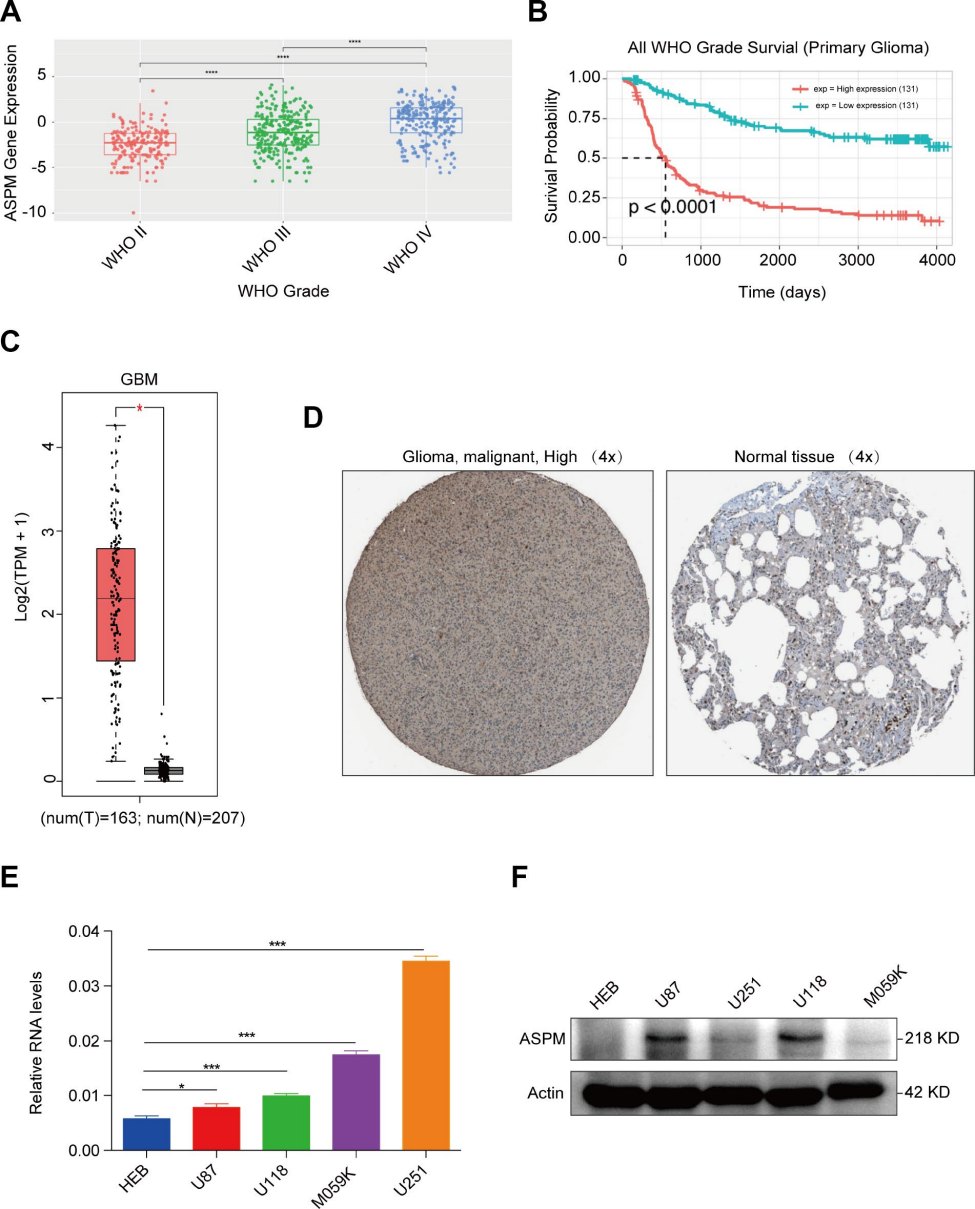
**ASPM is highly expressed in human GBM tissues and cell lines.** (**A**) Expression profiles of ASPM in GBM form grade II to grade IV. Red points indicate the expression level of ASPM in grade II glioma; green points indicate the expression level of ASPM in grade III glioma, and blue points indicates the expression level of ASPM in grade IV glioma. (**B**) Patients with high expression of ASPM in glioma had a significantly poor prognosis (n=262, p < 0.0001). (**C**) Box plot of ASPM expression in GBM from GEPIA. The box plot is based on 163 GBM cancer samples and 207 normal samples. The expression level of RNA was upregulated in GBM tissues compared with normal tissues. *p < 0.05 indicates a significant difference. (**D**) The Human Protein Atlas project shows representative immunohistochemical images of ASPM in GBM compared with normal tissues. (**E**) and (**F**) The RNA and protein levels of ASPM were detected in four GBM and one normal glial cell line by RT-qPCR and western blot. *p < 0.05, *** p < 0.001.

In our analysis, the upregulated gene with the highest fold change was found to be ASPM. We investigated the expression pattern of ASPM in cancer patients compared with that of normal samples using the GEPIA database (http://gepia.cancer-pku.cn) [10]. The results showed that ASPM is overexpressed in most human cancers, such as GBM, oesophageal carcinoma (ESCA), liver hepatocellular carcinoma (LIHC) and pancreatic adenocarcinoma (PAAD). In these cancers, ASPM acted as an oncogene. We also noted that ASPM may have a tumour suppressive effect in acute myeloid leukaemia (LAML), in which ASPM expression is lower in cancer tissues than in adjacent, normal tissues (Supplementary Figure 1). By exploring expression data from the Chinese Glioma Genome Atlas (CGGA) (http://www.cgga.org.cn), we found that the expression of ASPM increased with the increase of glioma grade (Figure 5A). Patients with high expression of ASPM in glioma had a significantly poor prognosis (n=262, p < 0.0001) (Figure 5B). Consistent with the results of CGGA, we found that ASPM transcription levels were significantly elevated in GBM tissues in TCGA (Figure 5C). By exploring clinical pathology data from the Human Protein Atlas project (https://www.proteinatlas. org) [11], we discovered that ASPM was moderately expressed in glioma tissues but weakly expressed in normal cerebral cortex specimens (Figure 5D). We next investigated the possible causes of ASPM upregulation in GBM and analysed the methylation level of ASPM transcriptional start site (TSS) from 2 kb upstream to 0.5 kb downstream. We did not observe significant differences in methylation levels (http://bio-bigdata.hrbmu.edu.cn/diseasemeth/index.html). Moreover, we found no significant correlation between gene expression and copy number gain (https://xena.ucsc.edu). (Supplementary Figures 2, 3). So, the mechanism of ASPM overexpression in GBM needs further study.

We then examined the expression of ASPM in glioma cell lines and discovered that the mRNA and protein expression levels of ASPM were similarly upregulated in 4 human glioma cell lines compared with the normal glial cell line (Figure 5E–5F). Taken together, these results collectively demonstrate that the upregulation of ASPM could serve as a new target for the investigation of potential GBM biomarkers.

### ASPM enhances tumorigenesis of GBM in vitro and in vivo

**Figure 6:**
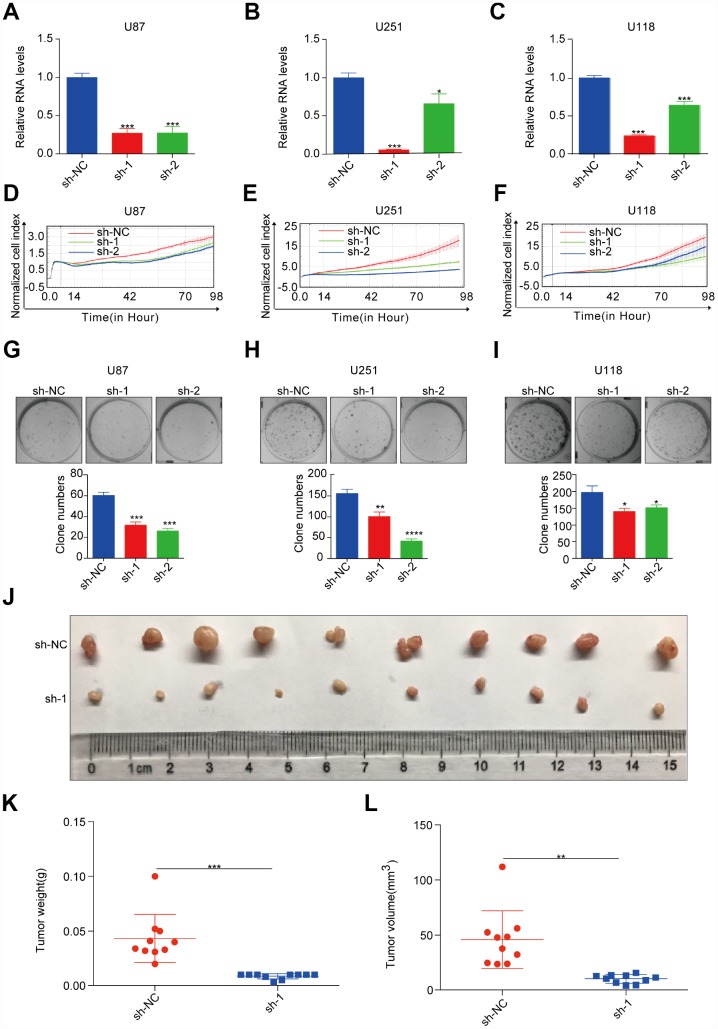
**ASPM enhances tumorigenicity of GBM cells *in vitro* and *in vivo*.** (**A**–**C**) RT-qPCR analysis of ASPM expression in stable knockdown U87, U251 and U118 cells. GAPDH was used as an internal reference. sh-NC: shRNA control, sh-1/2: shRNA targeting ASPM. *p < 0.05 versus control. (**D**–**F**) The growth rate of U87, U251 and U118 cells with stable ASPM knockdown was monitored by the RTCA-MP system. (**G**–**I**) Colony formation assay was performed using U87, U251 and U118 cells with stable ASPM knockdown. Representative images (up) and quantification analysis (down) are shown, and the data shown are the mean ± SD (n = 3). ***p < 0.001 versus control. (**J**–**L**) ASPM knockdown inhibited tumour growth in the U251 xenograft assay. Photos of xenograft tumours (**J**), tumour volume (**K**) and tumour weight (**L**) are shown. sh-NC, n = 10; sh-1, n = 10. **p < 0.01, ***p < 0.001 versus control.

To evaluate the oncogenic role of ASPM in GBM, we chose the human GBM cell lines U87, U251, and U118 to perform loss-of-function assays, as these lines express relatively higher levels of ASPM (Figure 6D, 6E). Next, we established stable ASPM knockdown cells using lentiviral shRNAs and identified those shRNAs that can readily knockdown ASPM expression (Figure 6A–6C). Silencing of ASPM resulted in a decrease in the cell proliferation of U87, U251 and U118 (Figure 6D–6F). Colony formation assays further demonstrated that ASPM knockdown significantly inhibited cell growth (Figure 6G–6I). To confirm the effect of ASPM on tumorigenesis in GBM, we performed a xenograft tumour formation assay to detect xenograft growth *in vivo*. U251 ASPM-sh-nc cells and ASPM-sh-1 cells were injected subcutaneously into nude mice. After 30 days, the volume and weight of subcutaneous tumours were calculated. Compared with the sh-NC cells, the volume and weight of xenograft tumours were significantly reduced (Figure 6J–6L). These results indicate the oncogenic activity of ASPM *in vivo*.

### Downregulation of ASPM could arrest the cell cycle of GBM cells and attenuate the Wnt/β-catenin signalling activity in GBM

**Figure 7:**
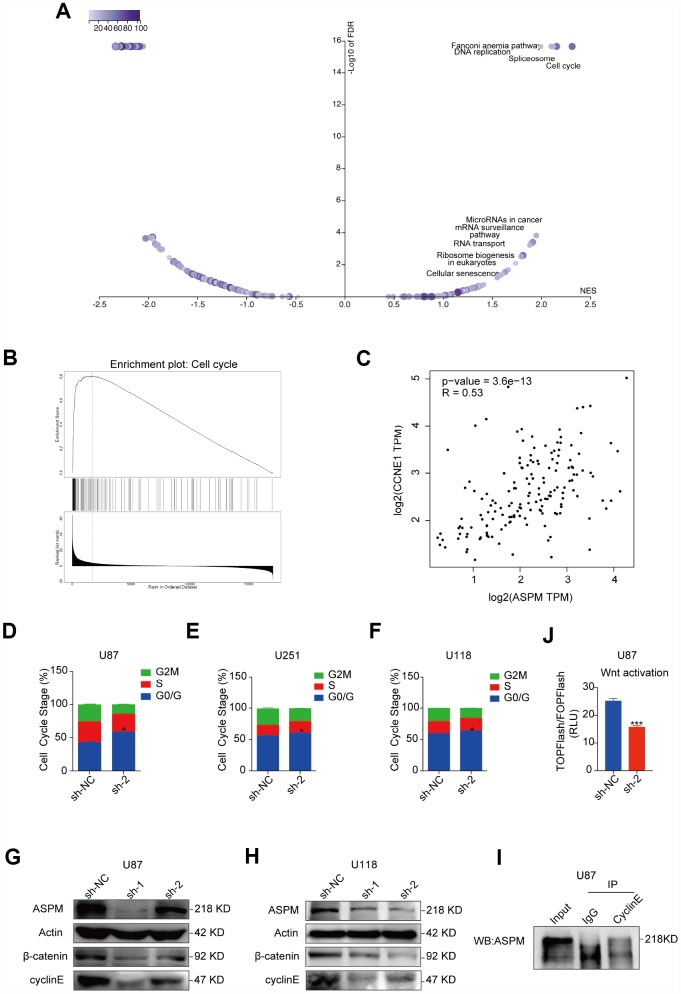
**Downregulation of ASPM could arrest the cell cycle of GBM cells at the G0/G1 phase.** (**A**, **B**) The enriched signalling pathways involved in ASPM-related genes by searching the LinkedOmics database. Cell cycle is the most enriched pathway. Volcano plot (**A**) and GSEA enrichment analysis (**B**) are shown. (**C**) Spearman’s correlation of the expression of cyclin E and ASPM in TCGA GBM, indicating a strong correlation. r = 0.53. (**D**–**F**) The cell cycle distribution of U87, U251 and U118 cells with stable ASPM knockdown was determined by PI staining and flow cytometry. Data shown are the mean ± SD (n = 3). G0/G1 phase distribution: *p < 0.05 versus control. (**G**, **H**) Western blot analysis of cyclin E and β-catenin protein levels in U87 and U118 cells with stable ASPM knockdown. (**I**) ASPM was immunoprecipitated with anti-cyclin E antibody from U87 cells, and the immunoprecipitates were subjected to Western blot analysis. (**J**) Luciferase activity of TOPFlash/FOPFlash in U87 cells that were transiently transfected with ASPM sh-NC or sh-2.

To further explore the underlying mechanism by which ASPM promotes the cell growth of GBM, we analysed the enriched signalling pathways involved in ASPM-related genes by searching the LinkedOmics (http://www.linkedomics.org/login.php) database and found that cell cycle is the most enriched pathway (Figure 7A). This is also supported by GSEA analysis (Figure 7B). It is known that ASPM could stabilize cyclin E abundance and passage through the G1 restriction point during NPC division [12], We observed that the expression of cyclin E is strongly correlated with ASPM in TCGA GBM by Spearman’s rank correlation (Figure 7C). Moreover, cell-cycle analysis revealed that stable knockdown of ASPM resulted in arrest at the G0/G1 phase in U87, U251 and U118 cells (Figure 7D–7F). As expected, knockdown of ASPM reduced the expression of cyclin E in U87 and U118 cells (Figure 7G, 7H). In addition, cyclin E was capable of co-immunoprecipitating ASPM (Figure 7I). These data indicate that ASPM interacts with cyclin E and is likely to control cell cycle via cyclin E.

Several studies have reported that ASPM acts as an essential regulator of the Wnt signalling process, which enhances tumorigenicity in cancers and facilitates neurogenesis in brain development [13–15]. Western blot analysis of U87, U251 and U118 cells ASPM knockdown significantly attenuated the levels of β-catenin (Figure 7G, 7H, Supplementary Figure 4). U87 cells were co-transfected with Wnt signalling reporter and ASPM shRNA encoding plasmids. Figure 7J shows that the luciferase activities were lower in ASPM knockdown cells, compared to the control cells. These findings indicate that ASPM enhance the Wnt/β-catenin signalling activity in GBM.

## DISCUSSION

Although a large number of basic and clinical studies have revealed the mechanisms of the formation and development of GBM in the past decades, the prognosis is still very poor and practically unchanged since 2000, when TMZ was introduced into clinical practice [16]. In the present study, using bioinformatics methods to deeply analyse the gene expression profiles of GSE14818, GSE22866 and GSE50161, a total of 362 DEGs, consisting of 185 upregulated and 177 downregulated DEGs, were screened between GBM and normal samples. Then, the DEGs were enriched into BP, CC and MF groups by GO terms, and signalling pathway enrichment was performed. Finally, we found that ASPM was overexpressed in GBM tissues and cell lines and enhanced GBM cell proliferation by promoting cell cycle progression *in vitro*. Xenograft growth suggests oncogenic activity of ASPM *in vivo*. Moreover, silencing of ASPM attenuated the levels of cyclin E and β-catenin.

Similar to our studies, recent studies have also reported the identification of DEGs in GBM. For example, Wang’s group analysed the GSE12657 and GSE42656 datasets consisting of 25 samples. Of these, 167 DEGs were identified, including 100 upregulated genes and 67 downregulated genes [17]. However, Wang’s research only contains 12 GBM and 13 normal brain samples, and most of the enriched pathways merely include one gene. In contrast to Wang's report, we used 3 datasets generated from 3 different races from the worldwide population. These indicated that our results are more reliable, but further analysis is still needed.

According to our analysis, one of the most notable genes was ASPM, which has the highest fold change among DEGs. Thus, it might be an oncogene that drives cancer cell growth in GBM. To our knowledge, ASPM has been shown to be a centrosomal protein associated with neurogenesis and brain size [18]. We found that ASPM is overexpressed in most human cancers. In GBM, we found the mRNA and protein expression levels of ASPM were significantly elevated in GBM tissues and GBM cell lines, same as Bikeye et al. conclusion [19]. As reported in many studies, it acts as an oncogene that is upregulated in some types of human cancers, including gastric cancer [20], prostate cancer [21], pancreatic cancer [13], ovarian cancer [22] and GBM [23]. In addition, we found that stable knockdown of ASPM resulted in a decrease in cell proliferation *in vivo* and *in vitro*. In 2010, Sanson et al. described that ASPM expression was positively correlated with tumour grade and increased with tumour recurrence, while silencing of ASPM resulted in dramatic proliferation arrest and cell death in glioma sphere models [19]. S. Horvath et al. indicated that ASPM knockdown inhibits tumour cell proliferation and neural stem cell proliferation [23]. Moreover, we discovered that stable knockdown of ASPM resulted in cell cycle arrest at the G0/G1 phase in U87, U251 and U118 cells and reduced the expression of cyclin E and β-catenin. Previous studies have reported that ASPM tune cyclin E ubiquitination and the G1 restriction point and acts as a positive regulator of the Wnt signalling process [12, 13, 15, 21]. These results collectively demonstrate that ASPM may serve as a new therapeutic target for GBM.

## CONCLUSIONS

Using three profile datasets and integrated bioinformatical analysis, 362 key candidate DEGs were identified, and GO and signalling pathway enrichment analysis showed that most of the key genes were significantly enriched in cell cycle. In our analysis, ASPM showed aberrantly high expression in GBM tissues and cell lines. Loss-of-function assay verified the oncogenic activity of ASPM *in vitro* and *in vivo*. Finally, downregulation of ASPM could arrest the cell cycle of GBM cells at the G0/G1 phase, and ASPM enhance the Wnt/β-catenin signalling activity in GBM. These findings could significantly enhance our understanding of the aetiology and underlying molecular events of GBM and suggest that ASPM may serve as a new target for the therapeutic treatment of GBM.

## MATERIALS AND METHODS

### Microarray data information

GBM and normal brain tissue gene expression profile datasets were downloaded from the National Center for Biotechnology Information (NCBI) GEO database, from which GSE14818, GSE22866 and GSE50161 were obtained. The GSE14818 microarray data were based on the GPL6370 Platform (Agilent Technologies, USA) and included 10 GBM tissues and 2 normal brain tissues (Submission date: Feb 13, 2009). The GSE22866 microarray data were based on the GPL4133 Platform (Agilent Technologies, USA) and included 40 GBM tissues and 6 normal brain tissues (Submission date: Jul 09, 2010). The GSE50161 microarray data were based on the GPL570 Platform (Affymetrix, USA) and included 130 tumour and normal brain tissues (Submission date: Aug 23, 2013). We selected 34 GBM tissues and 8 normal brain tissues from these data. Therefore, a total of 104 samples, including 88 cases of GBM samples and 16 cases of normal brain samples, were selected. We chose these 3 datasets for integrated analysis in this study because they contain a relatively large number of samples compared with other datasets, and these 3 datasets represented three different racial populations: GSE14181 was from Germany, GSE22866 was from France, and GSE50161 was from the United States. The original files and the platform probe annotation files were downloaded.

### Screening for DEGs

The raw GSE50161 data were classified into GBM and normal groups and analysed using R software (version 3.4.3). Additional software packages (affy, RCircos and pheatmap) were taken from the Bioconductor project (http://www.bioconductor.org/). Data analysis of the probe level data used a background-adjusted and normalized robust multi-array average (RMA) [24]. The normalized array data of the GSE14818 and GSE22866 datasets were downloaded from GEO directly, and each dataset was classified into GBM and normal brain groups. Student’s t-tests were conducted between GBM and normal brain samples. Statistically significant DEGs were defined with *p* < 0.05 and |log FC| > 1 as the cut-off criterion. The ID corresponding to the probe name was converted into the international standard name for genes. The list of DEGs from the three microarray datasets was saved as TXT files, and then the TXT format data were processed in Bioinformatics and Systems Biology Website (http://bioinformatics.psb.ugent.be/). A list of the genes that were up- or downregulated in the three microarrays was used for subsequent analysis.

### Validation of the DEGs in the TCGA GBM dataset

The TCGA database provides extensive genetic studies of human gene expression and specific disease associations. In the present study, we downloaded the TCGA GBM dataset to confirm the reliability of the identified DEGs using the same strategy as used in this report.

### GO and KEGG pathway enrichment analyses of DEGs

The functional enrichment of the validated DEGs was assessed based on the GO term and KEGG pathway annotations [25, 26]. GO term analyses were performed by using the DAVID database (https://david.ncifcrf.gov/), which is an essential foundation for the success of any high-throughput gene function analysis. Pathway analysis was carried out using multiple online databases, including DAVID and KEGG PATHWAY (http://www.genome.jp/kegg). *P*-values < 0.05 were considered statistically significant for GO term enrichment analysis and KEGG pathway analysis.

### Cell culture

Human U87, U251, and U118 glioma cell lines, a normal human astrocyte cell line (HEB) and a human embryonic kidney cell line (HEK-293) were cultured in high glucose Dulbecco’s modified Eagle medium (DMEM, Gibco, NY, USA) supplemented with 10% foetal bovine serum. All cells were incubated in a humidified incubator at 37 °C and 5% CO_2_. All cell lines were donated from Cancer Hospital Chinese Academy of Medical Sciences.

### RNA isolation and quantitative real-time PCR (qRT-PCR) assays

Total RNA was isolated from cultured cells using Trizol reagent (Invitrogen, USA) according to the manufacturer’s instructions. First-strand cDNA was generated using the PrimeScript^TM^II 1^st^ Strand cDNA Synthesis Kit (Takara, Dalian, China). Real-time PCR was performed in the StepOne™ Real-Time PCR System (Applied Biosystems, Foster City, USA) using TB Green™ Premix Ex Taq™ (Tli RNaseH Plus) (Takara, Dalian, China), and GAPDH was used as the internal control. The primers used are listed: ASPM sense: 5'- GAGACCTTGGTGGAATACCTGC-3', ASPM anti-sense: 5'- ACGAAGATCCAAAAGCCT TGCAC-3', GAPDH sense: 5'-GGGGTCATTG ATGGCAACAATA-3', GAPDH anti-sense: 5'-ATGG GGAAGGTGAAGGTCG-3'.

### Cell proliferation assay

The proliferation ability of glioma cells was monitored by using the xCELLigence Real-Time Cell Analyzer (RTCA)-MP system (Acea Biosciences/Roche Applied Science). This platform is able to measure cellular growth status in real time. First, 100 μl of culture medium was added to each well of E-Plate 96 (Roche Applied Science) to obtain equilibrium. Then, 2,000 cells in 100 μl of culture medium were seeded in each well. E-Plate 96 was locked in the RTCA-MP device at 37 °C with 5% CO_2_. Measured changes in electrical impedance were presented as a cell index that directly reflects cellular proliferation on biocompatible microelectrode coated surfaces. The cell index was read automatically every 15 min, and the recorded curve was depicted as the cell index ± SD.

### Colony formation assay

A total of 1 × 10^3^ cells per well were seeded into 6-well plates in triplicate. After culture for 10 days, each well was washed three times with phosphate buffered saline (PBS), fixed with methyl alcohol for 10 min, nd then stained with 1% crystal violet solution for 5 min. After washing, the colonies in each group were counted and imaged. Each experiment was repeated in triplicate.

### Cell cycle analysis

The cells were harvested and washed with PBS, adding 70% ice-cold ethanol at -20 °C overnight. Cells were then sequentially washed once in PBS and stained with 500 μl propidium iodide buffer (50 μg/ml PI, 50 μg/ml RNase A, 0.1% Triton) for 30 min at 37 °C and then immediately analysed using flow cytometry (BD Biosciences, San Jose, CA). The percentage of cells in different phases was counted and compared.

### Western blot analysis

Total cell lysates were prepared in RIPA lysis buffer (APPLYGEN, Beijing, China). Identical quantities of proteins were separated by sodium dodecyl sulfate-polyacrylamide gel electrophoresis (SDS-PAGE) and transferred onto PVDF membranes. After incubation with antibodies specific for ASPM (Proteintech, USA), cyclin E (Proteintech, USA), CDK2 (Santa, USA), β-catenin (Santa, USA) and β-actin (Sigma-Aldrich, USA), the blots were incubated with anti-rabbit IgG or anti-mouse IgG- conjugated to horseradish peroxidase (HRP) and detected using ImageQuant™ LAS 4000 (GE Healthcare Life Sciences, USA). β-actin was used as a loading control.

### Construction of stable cell lines with overexpression or downregulation of ASPM

ASPM shRNA-1 and shRNA-2 knockdown constructs were sub-cloned into the pLKO.1 puro cloning vector, and the shRNA sequences are shown: ASPM shRNA-1 5′CCGGCCAAAGTTGTTGACCGTATTTCTCGAGAAATACGGTCAACAACTTTGGTTTTTG-3′ and ASPM shRNA-2 5′-CCGGCGGCAATAAGTCGTC TTCAAACTCGAGTTTGAAGACGACTTATTGCCGTTTTTG -3′. They are synthesized by Generay (Shanghai, China). To produce lentiviral particles, HEK 293T cells were cultured overnight to reach approximately 80% confluence before transfection. Transfection was performed using the Lipofectamine 2000 reagent (Invitrogen, USA) according to the manufacturer’s instructions. Then, 5 μg of pLKO.1 shControl (sh-NC) or pLKO.1 sh-1 plasmid, 3.75 μg of psPAX2, and 1.25 μg of pVSV-G were introduced into the cells. After incubation for 48 h, culture medium containing the lentiviral particles was collected and filtered through a 0.45 μm filter to remove any remaining cells and debris. Target cells were infected for 24 h with lentiviral particles in the presence of 8 μg/ml polybrene and screened with puromycin to establish stable cells.

### In vivo xenograft model

Briefly, 4-week-old male athymic nude mice were used for the xenograft model. U251 cells stably expressing sh-NC and sh-1 were trypsinized and washed twice with sterilized PBS, and then 0.2 ml of PBS containing 5 x 10^6^ cells was subcutaneously inoculated into the inguinal area of the mice. Tumour volume was calculated using the formula: tumour volume = 0.5 × (width)^2^ × length. All animal experiments were undertaken in accordance with the NIH Guide for the Care and Use of Laboratory Animals, with the approval of the Institutional Animal Care and Use Committee of the Cancer Hospital Chinese Academy of Medical Sciences.

### Coimmunoprecipitation (co-IP)

U87 cells were lysed in RIPA (Beyotime, China) containing a protease inhibitor (Sigma-Aldrich, USA) and placed on ice for 30 min, vortex-mixed once every 10 min. The protein supernatant was collected by centrifugation at 12000 rpm in a centrifuge at 4 °C for 15 minutes. After determining the protein concentration, 30 μl of Protein A/G Agarose was added to the supernatant and then placed on the mixer for 4h at 4°C. After centrifugation at 3000 rpm for 5 minutes, the supernatant was transferred to a new tube. Then, the lysates were incubated with 10 μl of anti-cyclinE (Santa Cruz, USA) at 4 °C overnight with constant shaking. Next, 30 μl of Protein A/G Agarose were added to the sample and shaken slowly at 4 °C for 2 hours. Washing with protein lysate 6 times. The supernatant was discarded, and the pellet was resuspended by adding 30 μl 1 x protein loading buffer, and analysed by Western blot.

### Luciferase reporter assay

U87 cells (5×10^4^ cells per 24 well) were co-transfected with either the Wnt signaling reporter (TOPFlash or FOPFlash, Qualityard Bio, China), along with the ASPM shRNA encoding plasmids (sh-2 plasmid or sh-NC) 24 h after cell seeding using Lipofectamine 2000 (Invitrogen, USA). The ratio of Wnt signaling reporter/ASPM shRNA encoding plasmids was titrated (2: 1). The 50 ng pRL-TK vector (Qualityard Bio, China) that provided the constitutive expression of renilla luciferase was co-transfected as an internal control. After transfection for 48 h, the cells were harvested for analysis with the dual-luciferase reporter assay system (Promega, USA). Transfections were performed in quadruplicate and repeated at least three times in separate experiments. The transfection efficiency was normalized with renilla luciferase activity. The data are represented as normalized TOPFlash/FOPFlash values.

### Statistical analysis

Data are presented as the mean ± standard deviation (SD). Significance was determined using the two-tailed Student’s t test. Differences were deemed statistically significant at *p* < 0.05. *p < 0.05 and ** *p* < 0.01.

## Supplementary Material

Supplementary Figures

Supplementary Table 1

## References

[1] Mcneill K, Aldape K, Fine HA. Adult High-Grade (Diffuse) Glioma. 2015.

[2] Ostrom QT, Gittleman H, Xu J, Kromer C, Wolinsky Y, Kruchko C, Barnholtz-Sloan JS. CBTRUS Statistical Report: Primary Brain and Other Central Nervous System Tumors Diagnosed in the United States in 2009-2013. Neuro Oncol. 2016; 18:v1–v75. 10.1093/neuonc/now207 28475809 PMC8483569

[3] Ostrom QT, Gittleman H, Fulop J, Liu M, Blanda R, Kromer C, Wolinsky Y, Kruchko C, Barnholtz-Sloan JS. CBTRUS Statistical Report: Primary Brain and Central Nervous System Tumors Diagnosed in the United States in 2008-2012. Neuro Oncol. 2015; 17:iv1–iv62. 10.1093/neuonc/nov189 26511214 PMC4623240

[4] Seystahl K, Wick W, Weller M. Therapeutic options in recurrent glioblastoma—an update. Crit Rev Oncol Hematol. 2016; 99:389–408. 10.1016/j.critrevonc.2016.01.018 26830009

[5] Stupp R, Hegi ME, Mason WP, van den Bent MJ, Taphoorn MJ, Janzer RC, Ludwin SK, Allgeier A, Fisher B, Belanger K, Hau P, Brandes AA, Gijtenbeek J, Marosi C, Vecht CJ, Mokhtari K, Wesseling P, Villa S, Eisenhauer E, Gorlia T, Weller M, Lacombe D, Cairncross JG, Mirimanoff RO; European Organisation for Research and Treatment of Cancer Brain Tumour and Radiation Oncology Groups; National Cancer Institute of Canada Clinical Trials Group. Effects of radiotherapy with concomitant and adjuvant temozolomide versus radiotherapy alone on survival in glioblastoma in a randomised phase III study: 5-year analysis of the EORTC-NCIC trial. Lancet Oncol. 2009; 10:459–66. 10.1016/S1470-2045(09)70025-7 19269895

[6] Scott JN, Rewcastle NB, Brasher PM, Fulton D, MacKinnon JA, Hamilton M, Cairncross JG, Forsyth P. Which glioblastoma multiforme patient will become a long-term survivor? A population-based study. Ann Neurol. 1999; 46:183–88. 10.1002/1531-8249(199908)46:2<183::AID-ANA7>3.0.CO;2-7 10443883

[7] Service RF. Microchip arrays put DNA on the spot. Science. 1998; 282:396–99. 10.1126/science.282.5388.396 9841392

[8] Etcheverry A, Aubry M, de Tayrac M, Vauleon E, Boniface R, Guenot F, Saikali S, Hamlat A, Riffaud L, Menei P, Quillien V, Mosser J. DNA methylation in glioblastoma: impact on gene expression and clinical outcome. BMC Genomics. 2010; 11:701. 10.1186/1471-2164-11-701 21156036 PMC3018478

[9] Griesinger AM, Birks DK, Donson AM, Amani V, Hoffman LM, Waziri A, Wang M, Handler MH, Foreman NK. Characterization of distinct immunophenotypes across pediatric brain tumor types. J Immunol. 2013; 191:4880–8. 10.4049/jimmunol.1301966 24078694 PMC3827919

[10] Tang Z, Li C, Kang B, Gao G, Li C, Zhang Z. GEPIA: a web server for cancer and normal gene expression profiling and interactive analyses. Nucleic Acids Res. 2017; 45:W98–W102. 10.1093/nar/gkx247 28407145 PMC5570223

[11] Uhlen M, Zhang C, Lee S, Sjöstedt E, Fagerberg L, Bidkhori G, Benfeitas R, Arif M, Liu Z, Edfors F, Sanli K, von Feilitzen K, Oksvold P, et al. A pathology atlas of the human cancer transcriptome. Science. 2017; 357:eaan2507. 10.1126/science.aan2507 28818916

[12] Capecchi MR, Pozner A. ASPM regulates symmetric stem cell division by tuning Cyclin E ubiquitination. Nat Commun. 2015; 6:8763. 10.1038/ncomms9763 26581405 PMC5025044

[13] Pai VC, Hsu CC, Chan TS, Liao WY, Chuu CP, Chen WY, Li CR, Lin CY, Huang SP, Chen LT, Tsai KK. ASPM promotes prostate cancer stemness and progression by augmenting Wnt-Dvl-3-β-catenin signaling. Oncogene. 2019; 38:1340–53. 10.1038/s41388-018-0497-4 30266990

[14] Major MB, Roberts BS, Berndt JD, Marine S, Anastas J, Chung N, Ferrer M, Yi X, Stoick-Cooper CL, von Haller PD, Kategaya L, Chien A, Angers S, MacCoss M, Cleary MA, Arthur WT, Moon RT. New regulators of Wnt/beta-catenin signaling revealed by integrative molecular screening. Sci Signal. 2008; 1:ra12. 10.1126/scisignal.2000037 19001663

[15] Buchman JJ, Durak O, Tsai LH. ASPM regulates Wnt signaling pathway activity in the developing brain. Genes Dev. 2011; 25:1909–14. 10.1101/gad.16830211 21937711 PMC3185963

[16] Czapski B, Baluszek S, Herold-Mende C, Kaminska B. Clinical and immunological correlates of long term survival in glioblastoma. Contemp Oncol (Pozn). 2018; 22:81–85. 10.5114/wo.2018.73893 29628799 PMC5885076

[17] Wang A, Zhang G. Differential gene expression analysis in glioblastoma cells and normal human brain cells based on GEO database. Oncol Lett. 2017; 14:6040–44. 10.3892/ol.2017.6922 29113243 PMC5661398

[18] Kouprina N, Pavlicek A, Collins NK, Nakano M, Noskov VN, Ohzeki J, Mochida GH, Risinger JI, Goldsmith P, Gunsior M, Solomon G, Gersch W, Kim JH, et al. The microcephaly ASPM gene is expressed in proliferating tissues and encodes for a mitotic spindle protein. Hum Mol Genet. 2005; 14:2155–65. 10.1093/hmg/ddi220 15972725

[19] Bikeye SN, Colin C, Marie Y, Vampouille R, Ravassard P, Rousseau A, Boisselier B, Idbaih A, Calvo CF, Leuraud P, Lassalle M, El Hallani S, Delattre JY, Sanson M. ASPM-associated stem cell proliferation is involved in malignant progression of gliomas and constitutes an attractive therapeutic target. Cancer Cell Int. 2010; 10:1. 10.1186/1475-2867-10-1 20142996 PMC2817685

[20] Vange P, Bruland T, Beisvag V, Erlandsen SE, Flatberg A, Doseth B, Sandvik AK, Bakke I. Genome-wide analysis of the oxyntic proliferative isthmus zone reveals ASPM as a possible gastric stem/progenitor cell marker over-expressed in cancer. J Pathol. 2015; 237:447–59. 10.1002/path.4591 26178168 PMC5049620

[21] Wang WY, Hsu CC, Wang TY, Li CR, Hou YC, Chu JM, Lee CT, Liu MS, Su JJ, Jian KY, Huang SS, Jiang SS, Shan YS, et al. A gene expression signature of epithelial tubulogenesis and a role for ASPM in pancreatic tumor progression. Gastroenterology. 2013; 145:1110–20. 10.1053/j.gastro.2013.07.040 23896173

[22] Brüning-Richardson A, Bond J, Alsiary R, Richardson J, Cairns DA, McCormack L, Hutson R, Burns P, Wilkinson N, Hall GD, Morrison EE, Bell SM. ASPM and microcephalin expression in epithelial ovarian cancer correlates with tumour grade and survival. Br J Cancer. 2011; 104:1602–10. 10.1038/bjc.2011.117 21505456 PMC3101901

[23] Horvath S, Zhang B, Carlson M, Lu KV, Zhu S, Felciano RM, Laurance MF, Zhao W, Qi S, Chen Z, Lee Y, Scheck AC, Liau LM, et al. Analysis of oncogenic signaling networks in glioblastoma identifies ASPM as a molecular target. Proc Natl Acad Sci USA. 2006; 103:17402–07. 10.1073/pnas.0608396103 17090670 PMC1635024

[24] Irizarry RA, Hobbs B, Collin F, Beazer-Barclay YD, Antonellis KJ, Scherf U, Speed TP. Exploration, normalization, and summaries of high density oligonucleotide array probe level data. Biostatistics. 2003; 4:249–64. 10.1093/biostatistics/4.2.249 12925520

[25] Ashburner M, Ball CA, Blake JA, Botstein D, Butler H, Cherry JM, Davis AP, Dolinski K, Dwight SS, Eppig JT, Harris MA, Hill DP, Issel-Tarver L, et al, and The Gene Ontology Consortium. Gene ontology: tool for the unification of biology. Nat Genet. 2000; 25:25–29. 10.1038/75556 10802651 PMC3037419

[26] Kanehisa M. The KEGG database. Novartis Found Symp. 2002; 247:91–101. 10.1002/0470857897.ch8 12539951

