# Supplementary material for: ASPM promotes glioblastoma growth by regulating G1 restriction point progression and Wnt-β-catenin signaling
Source: Aging (Albany NY). 2020 Jan 6;12(1):224–41. doi: 10.18632/aging.102612 (PMC6977704; doi:10.18632/aging.102612)
Supplement: Supplementary Figures [file aging-12-102612-s002..pdf]

SUPPLEMENTARY FIGURES

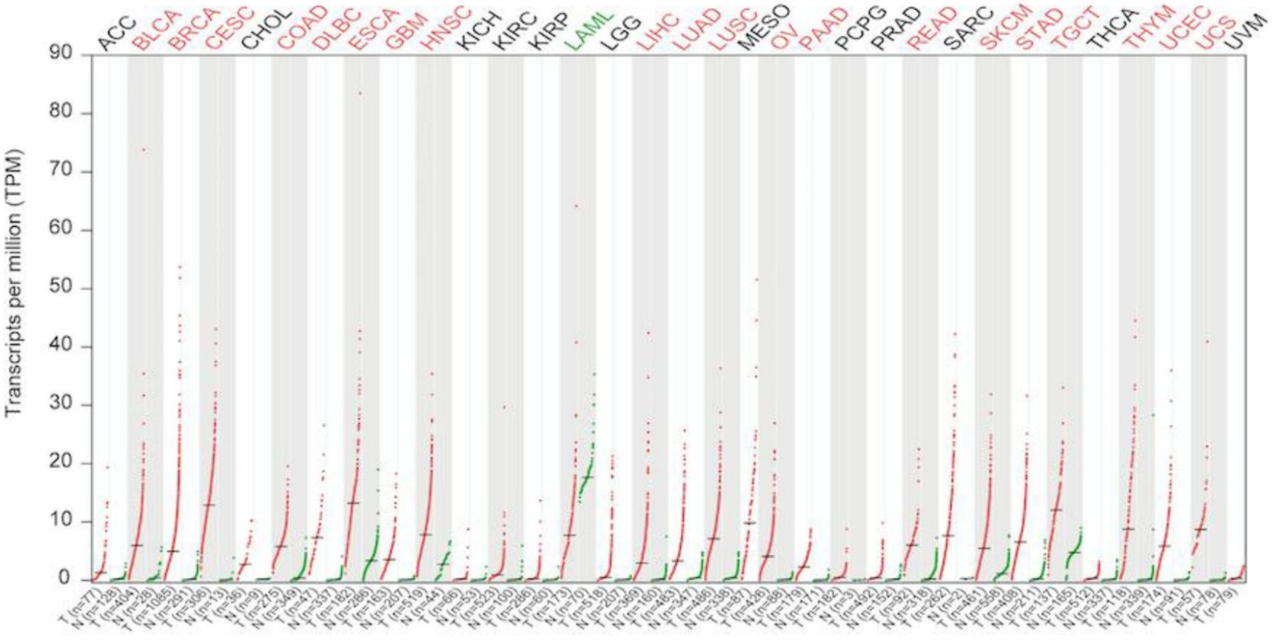

Supplementary Figure 1. Expression profiles of ASPM in all tumour types. At the top of the panel is the name of the cancer; red indicates that ASPM is highly expressed in the cancer tissue, black indicates that ASPM is not differentially expressed between cancer and normal tissues, and green indicates that ASPM is highly expressed in normal tissue.

diseaseName	genomic region	transcript	gene	p-value	meanMethylDisea-meanMethylNormal	plot
Glioblastoma multiforme[GBM]	chr1:197115324-197117824	NM_018136	aspm	6.149e-01	-0.004	-

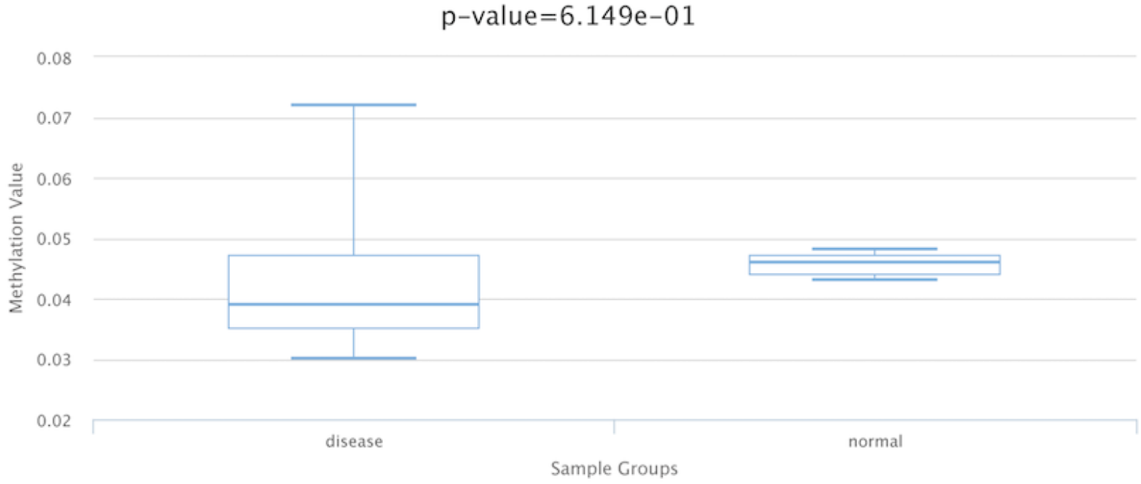

Supplementary Figure 2. The methylation level of ASPM transcriptional start site from 2 kb upstream to 0.5 kb downstream in GBM and normal tissue. There was no significant difference of methylation level between GBM and normal tissue.

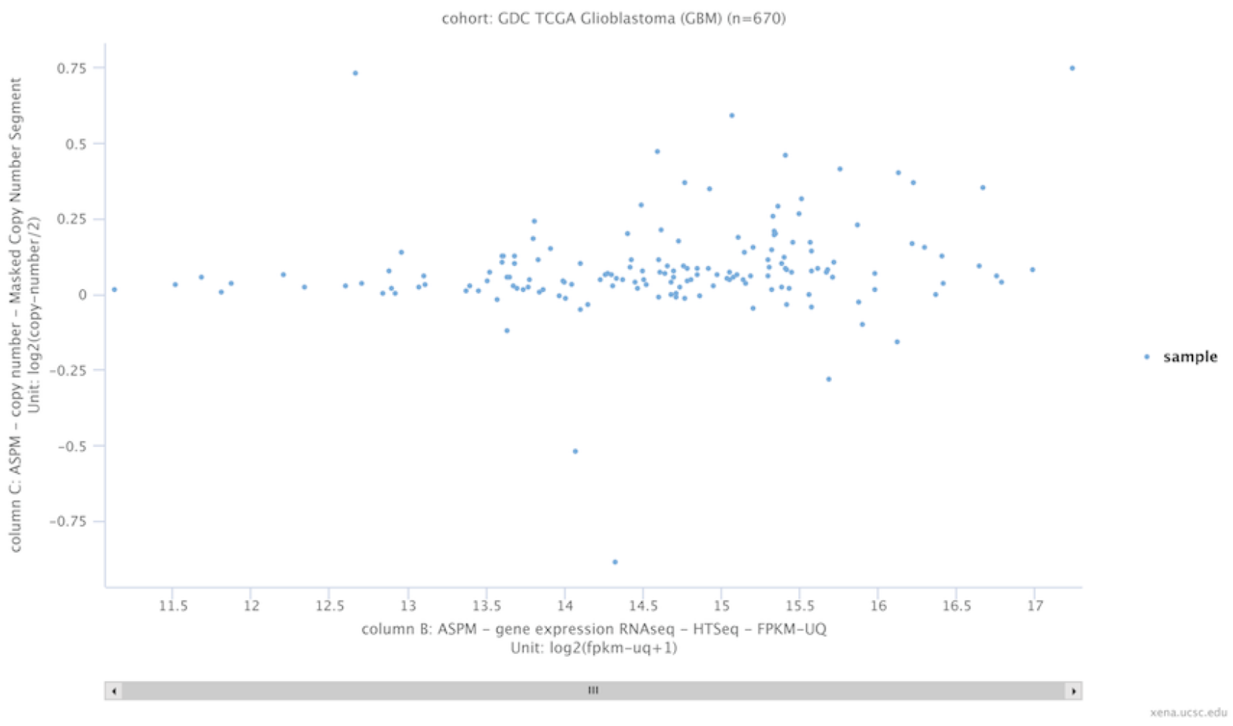

Supplementary Figure 3. The relationship between copy number of ASPM and its expression level in GBM.

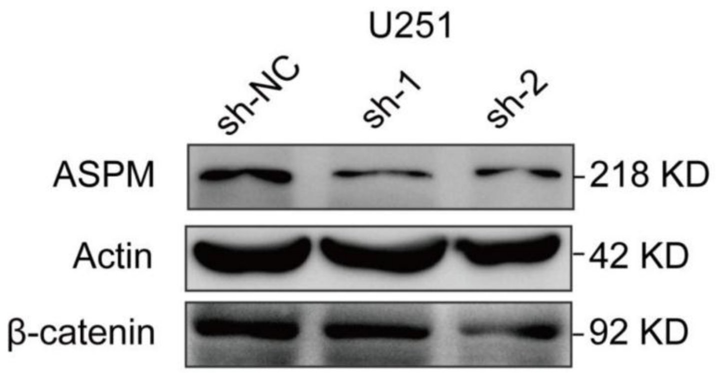

Supplementary Figure 4. Western blot analysis of ASPM and β-catenin protein levels in U251 cells with stable ASPM knockdown.
