# Supplementary material for: ASPM promotes glioblastoma growth by regulating G1 restriction point progression and Wnt-β-catenin signaling
Source: Aging (Albany NY). 2020 Jan 6;12(1):224–41. doi: 10.18632/aging.102612 (PMC6977704; doi:10.18632/aging.102612)
Supplement: Supplementary Table 1 [file aging-12-102612-s001..pdf]

SUPPLEMENTARY TABLE

Supplementary Table 1. Validation of the DEGs in TCGA dataset in GBM.

DEGs	
Up-regulated	MCM3 PPP4C HIST1H1C PDLIM5 TPX2 POLQ WDHD1<br>MOV10 CD276 ASPM ANXA2 PLAU MEOX2 NOX4 ANXA1 MMP2 CRISPLD1<br>SOAT1 BTN3A2 PDIA5 CDK2 SHOX2 VIM CTSC POLE2 ESPL1<br>CDC25A APOBEC3B CMTM6 CLEC2D KIF20A DEPDC1<br>HPSE HMGN1 LRRC17 RHOJ MPZL1 NEDD4 NEK2 TFAP2A SHC1<br>PLEKHG2 STK36 NUP205 TUBB6 TRAM2 CDKN2C RPS19 DTX3L<br>PAICS HMOX1 SDC1 ATAD2 KDELC1 TM9SF1 CASP2 CEP152<br>LSM5 RHOC ODC1 IL1RAP NECAP2 RNASEH2A DNAJC10<br>ELMOD2 CD44 FBXO9 FKBP14 GNS ARF4 KDELR2 PI4K2B<br>SLC39A1 GINS3 AK2 IKBKB TNFRSF11B DTYMK LIMS1<br>CPSF3 ETV6 TIMELESS PCNA C1orf112 SLC25A24 DKC1<br>TRAM1 MALT1 ODF2 PDIA6 SFT2D3 HMBS ARL6IP6 NIP7 DDX11<br>DCTN5 ZNF561 GUSB NME6 BAZ1A RSU1 RBMS1 TANC1 CASK ARMCX6<br>CLIC4 S100PBP TMED10 KLHL7 ITGB1 TWSG1 BRCA1 CENPN<br>MANEA RALA MCM7 KCTD3 REST PLOD3 ATP11C MND1 MTFR1<br>POLR2F COL4A1 EXO1 PLP2 LIMA1 FAM60A CASP8<br>TP53I3 CCNB1 CPVL RCC1 DBF4 KIF11 STIL GNG12 MASP1 GLIS3<br>RPLP0 ADAM17 ETV4 GNB2L1 CKAP2 RRM1 SNRPB RELA<br>RUVBL1
Down-regulated	EDNRA LIN7B COL18A1 SIRPA EIF4E3 SH3PXD2A RNF135 SERPING1<br>NTSR2 ELAVL3 CD99L2 KIF1B SCAMP1 ZNF540 CD47 BDH1<br>PDE4DIP ULK2 CX3CL1 EVL PRKCDBP CCNA1 FAM83H<br>RALGPS1 FLRT2 CTNNA2 OTUD1 NEBL ALDH5A1 CLDN10<br>AQP1 REPS2 GNG7 RGS20 APBB1 PRKCZ SNCA PDE1A CDH10 PPP2R2C<br>PGBD5 PARD6A SLC7A14 STOML1 CRY2 AKAP11<br>HABP4 INPP5A PLD3 CCDC92 YPEL2 FGFR3 SLC35F3 SELENBP1<br>FBXO44 PALM CYGB CLTB GABARAPL1 GHITM BCL11A<br>PREPL SYNGR1 MRPL41 ATP6V1H PTPRN SLC9A6 ARHGEF12<br>MPP2 FBXW7 OSBPL1A WNK2 EPHB6 CALM1 PTPRR ATP2A2 NRXN3<br>KNDC1 NAPB HPCA ATP13A2 RAP1GAP SYNJ1 BRWD1 AMPH<br>FXYD7 TEF MBP CHGB KCNH3 OLFM1 STXBP1 RGS7 PTPRT<br>BAIAP2 KCNB2 TMEM130 FBXL16 JPH1 STMN2 MYT1L EMX1 NELL1<br>CCK NSF EPHA10 PNMA3 HPRT1 SRGAP3 GABRB3<br>CYP4X1 AP3M2 SH3BGRL2 NECAP1 ARNT2 C1orf115<br>RAB11FIP4 STXBP6 GARNL3 GNAZ SH3GLB2 TYRO3<br>DDX24 KLHL2 PPP1R7
